# Death by the Bite of a Rattte-Snake

**Published:** 1848-07

**Authors:** 


*Death by the bite of a Rattte-snake.*—Our readers have doubtless read notices of the melancholy circumstances attending the death of the late Dr. WAINWRIGHT, of the city of New York, which occurred several months ago. In a late No. of the Annalist, we find the following report of the particulars of the case, as communicated to the New York Medical and Surgical Society.—EDITOR BUFFALO JOURNAL.

DR. POST mentioned some particulars of the late death in this city by the bite of a rattlesnake. The patient, a large well built man, 40 years of age, was bitten in the last phalanx of the middle finger of the left hand, near its articulation with the metacarpal bone. At the time the wound was made, a bystander observed that it was immediately followed by a small jet of blood. It was immediately sucked, and soon afterwards, less than half an hour, an attempt was made to excise it, and the excised surface was cauterised. . From the nature of the parts involved in the wound, the incision must have been very imperfect. A ligature was likewise tied firmly about the wrist, and 10 grs. Carb. Ammon. and ½ gr. Sulph. Morph. were administered. About 7½ P. M., the ligature, after it had been on half an hour, was removed. Previous to this time the hand had been very much swollen, but no swelling had occurred above the ligature. It now began to extend up the arm. At 9½ the patient was seen by Dr. P., and soon after by Dr. Parker; he had been visited by Dr. Cadwell previous to either of the other gentlemen being called upon. The swelling had now extended to a point half way between the elbow and shoulder-joint; it was very considerable, hard, and terminated abruptly—the finger, when passed along the arm, dropping suddenly from the swollen part to that in its natural condition. The hand was of a dark greenish colour, the lower part of the arm was mottled blue and greenish yellow; the discoloration did not extend as far as the swelling, and seemed to follow the swelling at about half an hour's interval.

At the time Dr. P. first saw the patient his pulse was 80, of medium fulness and strength, his face was flushed, and his manner excited. Half an hour afterwards the pulse began to flag, becoming less full and forcible, but increasing in frequency to 100; it afterwards reached 120 beats in the minute, but this was its maximum in frequency, becoming constantly more and more feeble. By 11 o'clock, the pulse was extinct at the wrist, but could still be felt at the groin. Between 10 and 11 he became stupid, taking no notice of what was passing about him. This lapsed into complete coma, and he died a little past 12. By this time the swelling had extended under the pectoral muscle, and the discoloration had reached the axilla.

The treatment, after Dr. P. saw the case, consisted in the administration of stimulants (brandy and Carb. Ammonia) in as large doses as the patient could be prevailed on to swallow. When they could no longer be given by the mouth they were administered by the rectum. It is somewhat remarkable that after the pulse had ceased at the wrist, and the surface had become covered with a cold perspiration, a sinapism applied to the epigastrium produced full redness in 25 minutes.

The snake had been brought from Alabama, and it was taken just before the accident from on shipboard, where it had been confined for a month. It was a large one, having 12 rattles, indicating it was 13 years old.

Dr. Adams stated, that in the cases which he had seen recorded a rapidly fatal result had only taken place in those in which a vessel seemed to be opened into.

Dr. Clark thought that a fatal result was exceedingly rare from the bite of snakes natives of the Eastern States, or of New York, states in which the rattlesnake never attains a very large size. In one instance, a man who kept these animals for exhibition, frequently having 50 in his possession, said he had been often bitten, but that he possessed a remedy which always cured him. He was at last bitten by a large one, and died in 4 hours. Dr. Clark stated, in experimenting with animals, he had frequently cauterised the bite of the animal immediately with Ammonia, entering the wound with a fine pointed syringe, and throwing in Aq. Ammon. Fort., but the animals (cats and rabbits) invariably died. The cat is affected in a singular manner by the bite of the rattlesnake, springing up convulsively 4 or 5 feet into the air, and on falling driving her talons forcibly into the floor; this would be repeated four or five times before the animal became insensible. In man the poison seemed to attack especially the subcutaneous cellular tissue; the sloughing which takes place when patients recover being superficial. From the discoloration following the swelling at so marked an interval it would seem that the skin was only secondarily attacked.

Several of the members alluded to the fact, that when a number of small animals were bitten by the same snake in quick succession, those bitten last generally escaped, whilst the first died. Dr. Metcalfe stated that, during his residence in Mississippi, though he had heard of a number of instances in which bites had been received, death had followed in only one case.

